# Clinical Application of Hemadsorption in Maintenance Hemodialysis Patients: An Adapted English Republication of the Chinese HA-MHD Consensus

**DOI:** 10.1016/j.xkme.2026.101528

**Published:** 2026-09-11

**Authors:** Xiaohua Sheng, Wei Lu, Zhiyong Guo, Shan Mou, Zhaohui Ni, Xiaoqiang Ding, Yueyi Deng, Guisen Li, Jun Liu, Pengli Luo, Shiren Sun, Huiming Wang, Jiali Wei, Xudong Xu, Xiangdong Yang, Zhenhua Yang, Wen Zhang, Jianrong Zhao, Xiaoling Zhou, Xiaoshuang Zhou, Zhu Zhou, Gengru Jiang, Niansong Wang, Nephrology Branch of the Shanghai Medical Association and the 2025 HA–MHD Consensus Working Group

**Affiliations:** 1 Department of Nephrology, Shanghai Sixth People's Hospital, Shanghai Jiao Tong University School of Medicine, Shanghai, China; 2 Department of Nephrology, Rheumatology and Immunology, Xinhua Hospital, Shanghai Jiao Tong University School of Medicine, Shanghai, China; 3 Department of Nephrology, First Affiliated Hospital of Naval Medical University, Shanghai, China; 4 Department of Nephrology, Renji Hospital, Shanghai Jiao Tong University School of Medicine, Shanghai, China; 5 Department of Nephrology, Zhongshan Hospital, Fudan University, Shanghai, China; 6 Department of Nephrology, Longhua Hospital, Shanghai University of Traditional Chinese Medicine, Shanghai, China; 7 Department of Nephrology, Sichuan Provincial People's Hospital, Chengdu, China; 8 Department of Nephrology, Shanghai General Hospital, Shanghai Jiao Tong University School of Medicine, Shanghai, China; 9 Department of Nephrology, Affiliated Hospital of Qinghai University, Xining, China; 10 Department of Nephrology, Xijing Hospital, Air Force Medical University, Xi'an, China; 11 Department of Nephrology, Renmin Hospital of Wuhan University, Wuhan, China; 12 Department of Nephrology, Hainan General Hospital, Haikou, China; 13 Department of Nephrology, Minhang Hospital, Fudan University, Shanghai, China; 14 Department of Nephrology, Qilu Hospital, Shandong University, Jinan, China; 15 Department of Nephrology, First Affiliated Hospital of Guangxi Medical University, Nanning, China; 16 Department of Nephrology, Ruijin Hospital, Shanghai Jiao Tong University School of Medicine, Shanghai, China; 17 Department of Nephrology, Affiliated Hospital of Inner Mongolia Medical University, Hohhot, China; 18 Department of Nephrology, General Hospital of Ningxia Medical University, Yinchuan, China; 19 Department of Nephrology, Shanxi Provincial People's Hospital, Taiyuan, China; 20 Department of Nephrology, First Affiliated Hospital of Kunming Medical University, Kunming, China

**Keywords:** hemadsorption, hemoperfusion, maintenance hemodialysis, protein-bound uremic toxins, expert consensus

## Abstract

The accumulation of protein-bound uremic toxins and middle- to large-molecular-weight toxins in patients receiving maintenance hemodialysis (MHD) results in long-term complications. Hemadsorption (HA) can efficiently remove these uremic toxins and reduce the risk of long-term complications in MHD patients. The Chinese HA-MHD Consensus Working Group has revised and updated the 2021 Shanghai Expert Consensus on the Clinical Application of Haemoadsorption in Maintenance Haemodialysis Patients to further standardize clinical application of HA and reflect the latest evidence. Based on an extensive literature search, the Grading of Recommendations Assessment, Development and Evaluation framework, and the modified Delphi method, the working group formulated 27 evidence-based recommendations regarding patient populations, session frequency, treatment modalities, precautions, and adverse reactions. This consensus systematically delineates the clinical thresholds and rationales for utilizing HA across three core dimensions: routine treatment regimens, intensive treatment regimens, and long-term complication risk management to provide comprehensive guidance. Furthermore, the consensus provides detailed guidance on individualized operational parameters to maximize both safety and treatment efficacy. This consensus aims to provide the latest guiding opinions for the rational and standardized application of HA therapy in patients receiving MHD.

## Introduction

Kidney failure is one of the primary public health challenges faced globally. The latest data from the China National Renal Data System show that as of the end of 2024, there were 1.027 million patients receiving maintenance hemodialysis (MHD) in China. The average dialysis vintage in the past 10 years has significantly increased from 46.7 months to 52.2 months, with approximately 10% of patients having a dialysis vintage of more than 10 years.^1^ As the dialysis vintage increases, long-term complications such as pruritus, sleep disorders, and cardiovascular diseases have become increasingly prominent in patients receiving MHD and are key constraints affecting patient quality of life and long-term prognosis.^2^

The insufficient clearance of protein-bound uremic toxins (PBUTs) and middle- to large-molecular-weight toxins in MHD patients is one of the core pathophysiological mechanisms leading to these complications. Traditional blood purification methods, such as low-flux hemodialysis (LFHD), high-flux hemodialysis (HFHD), and hemodiafiltration (HDF), rely primarily on diffusion and convection to remove toxins, but their ability to clear PBUTs and middle- to large-molecular-weight toxins is limited.^3^^,^^4^ Hemadsorption (HA) technology, with its unique adsorption mechanism, provides a critical strategy for clearing PBUTs and middle- to large-molecular-weight toxins that are difficult to remove effectively with conventional dialysis.^5^^,^^6^ Existing clinical evidence shows that combining HA with conventional hemodialysis (HAHD) can improve severe uremic pruritus, microinflammatory states, and sleep disorders in patients receiving MHD and can improve their quality of life and long-term survival.^7^, ^8^, ^9^, ^10^, ^11^

The *Shanghai Expert Consensus on the Clinical Application of Haemoadsorption in Maintenance Haemodialysis Patients* (2021 Edition) has played a positive role in promoting the standardized clinical application of HA technology and has been widely recognized by the nephrology community in China and worldwide. Based on the clinical perceptions of the consensus group, the use of HAHD in China has become increasingly widespread since 2021.^12^ With the emergence of high-quality evidence, several studies have further evaluated HAHD; notably, a multicenter randomized controlled trial (RCT) demonstrated significant reductions in the levels of targeted uremic toxins, and a large prospective cohort study observed an independent association between regular HA and improved survival.^7^^,^^10^ Driven by this new evidence and accumulating clinical experience, the working group has revised the 2021 edition to further enhance its guidance and practicality.

This consensus was revised under the guidance of the expert consensus working group. The working group systematically searched domestic and international databases such as CNKI, Wanfang, PubMed, Embase, and Google Scholar to identify evidence-based medicine for this consensus. The search terms included “haemoperfusion”, “haemoadsorption”, “maintenance haemodialysis”, “end-stage renal disease”, “uraemic toxins”, “uraemic complications”, and “protein-bound uraemic toxins”. The search period was up to June 30, 2025. The working group used the Grading of Recommendations Assessment, Development and Evaluation (GRADE) system to grade the quality of evidence and the strength of the recommendations (Tables 1 and 2). Moreover, based on the modified Delphi method, discussions were held in 3 offline meetings, and 23 experts participated in anonymous voting to reach consensus on the recommendations. The voting options were “agree”, “substantially agree”, “disagree”, and “uncertain”. The consensus rate was calculated as (agree + substantially agree)/total votes × 100%. Only when each recommendation received more than 75% “agree” or “substantially agree” votes was it included in this expert consensus. This consensus formed 27 recommendations, all of which were based on the best available current clinical research evidence. In addition, to ensure that patients receive the optimal efficacy and safety, clinical practice must integrate the evidence cited in this consensus with the patient’s individual situation to make rational clinical decisions. Importantly, this document represents an adapted and updated English republication of the Chinese expert consensus on hemadsorption, which was originally published in Chinese in the Shanghai Medical Journal in 2025.^13^ Although the core clinical recommendations remain strictly consistent with the primary version, this international edition has been significantly refined following rigorous peer review.

**Table 1:** GRADE Evidence Quality Levels

Evidence Level	Standard
A (High)	Further research is unlikely to change the confidence in the estimate of effect. Very confident that the true effect lies close to the estimate of effect.
B (Moderate)	Further research is likely to have an important impact on confidence in the estimate of effect and may change the estimate. Moderately confident in the effect estimate; the true value is likely to be close to the estimate, but there is still a possibility that they are substantially different.
C (Low)	Further research is very likely to have an important impact on confidence in the estimate of effect and is likely to change the estimate. Confidence in the effect estimate is limited; the true value may be substantially different from the estimate.
D (Very Low)	Any estimate of effect is very uncertain. Almost no confidence in the effect estimate; the true value is likely to be substantially different from the estimate.

**Table 2:** GRADE Recommendation Strength

Recommendation Strength	Description
Strong	Clearly shows that the benefits of the intervention outweigh the risks, or vice versa.
Weak	Benefits and risks are uncertain or closely balanced, regardless of the quality of evidence.

## Overview of HA

HA is a blood purification technique that introduces the patient’s blood into an extracorporeal circuit, passing it through a cartridge containing solid-phase adsorbents to selectively or nonselectively remove endogenous or exogenous pathogenic substances from the blood by physical adsorption, chemical binding, or biological affinity.^14^ The basic principle of HA is adsorption, ie, the use of a large specific surface area of the adsorbent to bind target molecules.

The cartridge consists of adsorbents and packaging materials, of which the adsorbent is key to achieving toxin clearance. Commonly used adsorbent materials in MHD patients include resins, activated charcoal, and polysaccharides (Table 3). In recent years, adsorbent material technology has developed rapidly. Nanoscale pore-adjusting technology and hypercrosslinking technology achieve precise control over the pore size, pore volume, and strength of novel adsorbent resin materials. Hypercrosslinking technology effectively prevents particle shedding and ensures the stability of the adsorption process by increasing the hardness of the resin and preventing its brittleness.^15^ The new generation of functionalized resins combines microcontrolled positive-charge technology with hypercrosslinking technology. By strengthening electrostatic forces, it synergizes with van der Waals forces, hydrophobic interactions, and pore-sieving effects, not only achieving relatively specific adsorption of PBUTs but also maintaining good adsorption performance for middle- to large-molecular-weight toxins.^16^

**Table 3:** Commonly Used Adsorbent Materials in MHD

Material Category	Common Material Types	Adsorption Mechanism	Adsorption Classification	Typical Adsorbed Substances	Representative Cartridges
Resin	Neutral macroporous resin (eg, styrene-divinylbenzene copolymer)	Van der Waals forces, hydrophobic interaction, 3D network molecular sieve effect	Broad-spectrum adsorption	PBUTs, middle-to-large molecular toxins, inflammatory mediators	HA130, HA230, KHA130, KHA200
	Functionalized resin (eg, grafted with positive charge groups)	Van der Waals forces, hydrophobic interaction, 3D network molecular sieve effect, and electrostatic interaction	Relatively specific adsorption	PBUTs, middle-to-large molecular toxins, inflammatory mediators	pHA130
Activated Charcoal	Spherical activated charcoal	Van der Waals forces, hydrophobic interaction	Broad-spectrum adsorption	Small molecular toxins (eg, creatinine, uric acid, guanidines, phenols, indoles)	Not intended for routine use in kidney failure
Polysaccharide	Porous cellulose microspheres (eg, grafted with hexadecyl chains)	Hydrophobic interaction, 3D network molecular sieve effect	Relatively specific adsorption	β_2_-microglobulin, inflammatory mediators	Lixelle S15, S25, S35

## Appropriate Populations and Treatment Frequencies

### Routine treatment regimen

Patients receiving MHD can routinely receive HA treatment. When patients develop the following complications, an individualized HA session frequency and duration should be prescribed according to symptom severity.

#### Chronic kidney disease-associated pruritus

**Recommendation 1:** We recommend routine screening and systematic assessment for chronic kidney disease-associated pruritus (CKD-aP) in MHD patients using standardized scale tools for severity grading (evidence quality: B; recommendation strength: strong; consensus level: 100%).

**Recommendation 2:** For MHD patients with CKD-aP, particularly those with a modified Duo's pruritus score >12 or a visual analog scale (VAS) score ≥7, we recommend once-weekly HA treatment in addition to dietary control, medication, and lifestyle adjustments (evidence quality: B; recommendation strength: strong; consensus level: 100%).

The diagnosis of CKD-aP must follow a standardized process. Following a detailed medical history and thorough physical examination, other primary skin diseases or systemic diseases that may cause pruritus must be excluded. Assessment should be conducted using standardized scale. Validated scoring systems establish critical thresholds for clinical intervention: a modified Duo's score > 12 or a VAS score ≥ 7 accurately identifies MHD patients with severe pruritus.^17^^,^^18^

The accumulation of inflammatory factors and PBUTs in MHD patients is closely related to the occurrence of CKD-aP. Research has shown that these toxins, through complex immune–nervous system interactions, are among the core pathophysiological factors that cause refractory pruritus in up to 80% of MHD patients, severely impairing their quality of life.^19^ The results from a prospective randomized crossover trial of 40 MHD patients demonstrated that serum interleukin (IL)-31 levels were significantly higher in MHD patients with CKD-aP than in those without, and the level of this inflammatory factor was positively correlated with pruritus severity. In terms of treatment, after HD combined with HA130 was used, the serum IL-31 clearance rate was 34.26 ± 1.43%, which was significantly higher than that in patients treated with HD alone (15.28 ± 2.11%, *P* < 0.01), suggesting that ameliorating pruritus by removing inflammatory mediators such as IL-31 may be a potential mechanism of action for HA.^20^ Furthermore, a prospective RCT involving 438 MHD patients from 37 dialysis centers nationwide demonstrated that compared with patients treated with LFHD or HFHD alone, patients treated with LFHD or HFHD combined with once-weekly HA130 showed a greater than 50% reduction in their modified Duo's pruritus score after 1 year.^7^ A recent systematic review further confirmed that among existing blood purification technologies, including HD, HFHD, and HDF, HA is the technique with the most sufficient evidence for ameliorating CKD-aP.^17^

#### Microinflammatory state

**Recommendation 3:** We recommend routine screening and systematic assessment for microinflammation in MHD patients by monitoring relevant inflammatory markers to identify high-risk patients (evidence quality: B; recommendation strength: strong; consensus level: 100%).

**Recommendation 4:** After infection, malignancy, and active rheumatic diseases are excluded, for MHD patients with a persistent microinflammatory state, particularly those with hs-CRP >3 mg/L, CRP >8 mg/L, IL-6 >16 pg/mL, or TNF-α >41 pg/mL, we recommend once-weekly HA treatment **(evidence quality: B; recommendation strength: strong; consensus level: 100%).**

The microinflammatory state is a common pathological process in MHD patients, denoting a chronic, low-grade systemic inflammatory response driven by elevated levels of proinflammatory cytokines (such as IL-6 and TNF-α) and acute-phase proteins (such as CRP), rather than a traditional acute infection.^21^ It is not only directly related to malnutrition, cardiovascular events, and mortality but is also a key factor driving the increased production of various uremic toxins, such as β_2_-microglobulin (β_2_-MG), thus forming a vicious toxin–inflammation cycle.^21^ Validated biomarkers establish critical thresholds for clinical intervention: a high-sensitivity C-reactive protein (hs-CRP) level > 3 mg/L, a CRP level > 8 mg/L, an IL-6 level > 16 pg/mL, or a tumor necrosis factor (TNF)-α level > 41 pg/mL can be used to accurately identify MHD patients with severe microinflammation.^22^, ^23^, ^24^, ^25^ The results from an RCT involving 92 MHD patients showed that HD combined with KHA200 treatment was significantly superior to HD alone in reducing IL-6 levels in MHD patients (IL-6 clearance rate: 24.73% vs 3.12%, *P* < 0.001).^15^ An RCT comparing the effects of 4 dialysis modalities (HD, HFHD, HDF, and HAHD) combined with low-calcium dialysate showed that once-weekly HD combined with HA130 was most effective in reducing IL-6, hs-CRP, and TNF-α levels.^26^ An RCT of 150 elderly (≥60 years) MHD patients demonstrated that after 6 months of once-weekly HD combined with HA130 treatment, the patients’ hs-CRP, homocysteine, IL-6, and TNF-α levels were significantly lower than those in the HD alone group, and their quality of life was significantly improved.^8^

#### Chronic kidney disease-mineral and bone disorder

**Recommendation 5:** We recommend routine screening and systematic assessment for chronic kidney disease-mineral and bone disorder (CKD-MBD) in MHD patients, including regular monitoring of biochemical indicators such as intact parathyroid hormone (iPTH) and timely imaging, to identify cardiovascular calcification risk (evidence quality: B; recommendation strength: strong; consensus level: 100%).

**Recommendation 6:** For MHD patients with CKD-MBD, particularly those with moderate-to-severe secondary hyperparathyroidism uncontrolled by medication (iPTH persistently > 600 pg/mL), or those with coronary artery calcification (Agatston score > 100 AU on cardiovascular computed tomography [CT]) or abdominal aortic calcification (Kauppila score > 4.5 on abdominal CT or lateral lumbar radiography), we recommend once-weekly HA treatment in addition to standard CKD-MBD management, including dietary control and the use of phosphate binders, vitamin D and its analogues, and calcimimetics (evidence quality: B; recommendation strength: strong; consensus level: 100%).

CKD-MBD is one of the most common systemic complications in MHD patients and is characterized by the occurrence of mineral metabolism disorders, bone remodeling abnormalities, and cardiovascular and soft tissue ectopic calcification driven by the accumulation of uremic toxins such as iPTH and fibroblast growth factor 23. It is a major driver of the significantly increased incidence of cardiovascular events and mortality in patients.^27^^,^^28^ Integrating mineral biomarkers (iPTH, calcium, and phosphorus) with vascular imaging guides individualized HA therapy. Validated scoring systems provide critical thresholds for intensified intervention: an Agatston score > 100 AU indicates an 8.5-fold higher risk of mortality, whereas a Kauppila score > 4.5 predicts a >2-fold higher risk of all-cause and cardiovascular mortality.^29^, ^30^, ^31^

Compared with HD alone, HD combined with a KHA200 cartridge significantly improved iPTH clearance (61.14% vs 30.21%, *P* < 0.001).^15^ A prospective cohort study of 93 MHD patients indicated that compared with HD alone, HD combined with HA130 treatment for 3 years significantly reduced patient PTH levels, improved bone pain symptoms, and decreased cardiovascular-related mortality (4.3% vs 17.0%, *P* = 0.049).^32^ A multicenter RCT of 438 MHD patients showed that a HFHD or LFHD combined with once-weekly HA130 could significantly reduce iPTH levels in MHD patients.^7^ The results from an RCT of 192 MHD patients demonstrated that with the use of low-calcium dialysate, once-weekly HD combined with HA130 treatment showed decreased iPTH and alkaline phosphatase levels, increased 25-hydroxyvitamin D levels, and more effectively reduced the incidence of cardiovascular calcification, with better effects than HD, HFHD, or HDF alone.^26^ A prospective controlled study involving 207 MHD patients showed that after 6 months of HD combined with HA130 treatment, patients’ carotid intima-media thickness and plaque area did not significantly increase, whereas these metrics tended to increase in the HD alone group.^33^

#### High β_2_-microglobulin levels

**Recommendation 7:** We recommend routine screening and systematic assessment of serum β_2_-MG levels in MHD patients to identify the risks of high-level accumulation and related complications (evidence quality: B; recommendation strength: strong; consensus level: 100%).

**Recommendation 8:** For MHD patients with persistently elevated β_2_-MG levels (>30 mg/L) or with dialysis-related amyloidosis, such as carpal tunnel syndrome, we recommend HA treatment 1-3 times per week (evidence quality: B; recommendation strength: strong; consensus level: 100%).

In MHD patients, the long-term accumulation of β_2_-MG is not only the core pathogenic protein of dialysis-related amyloidosis, but its elevated serum level has also been confirmed to be significantly associated with increased risks of cardiovascular disease, infection, fractures, and all-cause mortality.^34^^,^^35^ Routine monitoring of serum β_2_-MG levels is recommended to optimize blood purification strategies. Maintaining predialysis β_2_-MG levels < 30 mg/L significantly improves survival; thus, levels > 30 mg/L establish a critical threshold warranting intensified intervention.^35^

A multicenter RCT showed that HFHD or LFHD combined with once-weekly HA130 could significantly reduce serum β_2_-MG levels in MHD patients.^7^ A prospective study of 17 patients with dialysis-related amyloidosis indicated that after 1 year of thrice weekly HD combined with Lixelle S15 HA, patients’ serum β_2_-MG levels significantly decreased from 29.3 ± 9.6 mg/L to 24.7 ± 5.1 mg/L, whereas their activities of daily living scores increased significantly.^36^ Kinetic studies of β_2_-MG by Ronco et al^21^ showed that after an initial period of high-frequency HA treatment (3×/week), the overall β_2_-MG burden in the body was significantly reduced; subsequently, even with a lower frequency (1×/week) maintenance treatment, patients could still maintain a low average β_2_-MG level. Based on these findings, Ronco et al^21^ proposed the inflammation mitigation hypothesis to explain the long-term efficacy of HA; that is, insufficient clearance of β_2_-MG and other toxins in uremic patients leads to persistent subclinical inflammation, which in turn further stimulates the production of β_2_-MG. Therefore, HA not only effectively removes β_2_-MG and other middle- to large-molecular-weight inflammatory mediators from the blood but also further inhibits the generation of β_2_-MG and other inflammatory mediators by reducing the systemic inflammatory response.

#### Protein energy wasting

**Recommendation 9:** We recommend screening and systematic assessment for protein energy wasting (PEW) in MHD patients using standardized nutritional assessment tools (evidence quality: B; recommendation strength: strong; consensus level: 100%).

**Recommendation 10:** For MHD patients with PEW, particularly those with a modified quantitative subjective global assessment (MQSGA) score > 20 or malnutrition-inflammation score (MIS) > 18, we recommend once-weekly HA treatment in addition to targeted nutritional support (evidence quality: B; recommendation strength: strong; consensus level: 100%).

PEW is among the most significant complications in MHD patients. Its core pathological mechanism involves the accumulation of uremic toxins and the persistent microinflammatory state they induce. These two factors interact, causing anorexia, increased catabolism, and hindered anabolism, ultimately leading to the progressive depletion of the body's protein and energy stores. It is among the most effective independent predictors of hospitalization and mortality.^37^ Routine screening using validated nutritional assessment tools such as the MQSGA score or the MIS in clinical practice can aid in the early detection of malnutrition in MHD patients.^38^^,^^39^ Validated scoring systems establish critical thresholds for clinical intervention: an MQSGA score > 20 or an MIS > 18 accurately identifies hemodialysis patients suffering from severe malnutrition or PEW.^38^^,^^39^

A study showed that after 12 weeks of once-weekly HD+HA130 treatment, the body mass index of patients with end-stage diabetic nephropathy increased from 21.98 ± 2.28 kg/m^2^ to 24.30 ± 1.51 kg/m^2^, the hemoglobin concentration increased from 103.98 ± 12.76 g/L to 113.31 ± 12.94 g/L, and the albumin concentration increased from 32.75 ± 4.38 g/L to 35.73 ± 3.71 g/L; all these improvements were significantly greater than those in the HD and HDF groups.^40^ An RCT of 83 CKD-MBD patients showed that compared with 1-2×/month HA treatment, 4×/month HA treatment for 6 months resulted in significantly lower MQSGA scores and more significant improvements in body weight, arm muscle circumference, and calf circumference (*P* < 0.01), suggesting that a higher frequency of HA treatment more effectively ameliorated PEW.^41^ A prospective multicenter cohort study from Guizhou Province (4,623 MHD patients) showed that receiving HAHD treatment was a protective factor against PEW (odds ratio [OR] = 0.51, 95% confidence interval [CI] 0.31-0.87; *P* = 0.012).^10^ Multivariable Cox regression analysis showed that HA frequencies of 2×/month (hazard ratio [HR] = 0.40; *P* = 0.037), 3×/month (HR = 0.44; *P* = 0.014), and 4×/month (HR = 0.54; *P* = 0.008), as well as HA130 (HR = 0.57; *P* = 0.014) and HA230 (HR = 0.30; *P* = 0.001) cartridge types, were protective factors against PEW in MHD patients.

#### Uremia-Related Sleep Disorders

**Recommendation 11:** We recommend screening and systematic assessment of sleep quality in MHD patients using standardized questionnaires (evidence quality: B; recommendation strength: strong; consensus level: 96%).

**Recommendation 12:** For MHD patients with sleep disorders, particularly those with a Pittsburgh Sleep Quality Index (PSQI) ≥ 10, we recommend once-weekly HA treatment. For patients with a 5 < PSQI < 10, we recommend HA treatment once weekly or once every 2 weeks (evidence quality: B; recommendation strength: strong; consensus level: 100%).

Uremia-related sleep disorders are extremely common in MHD patients. Their occurrence is closely related to nervous system dysfunction caused by uremic toxins. They not only severely impair quality of life but are also independent risk factors for increased cardiovascular events and all-cause mortality.^42^ Routine assessment using standardized tools such as the PSQI can aid in the discovery and quantification of patients’ sleep problems.

A 2-year prospective cohort study of 158 MHD patients showed that compared with HD alone, HD combined with 1-2× HA130 every 2 weeks significantly prolonged sleep duration and improved sleep efficiency.^9^ After 3 months of once-weekly HD+HA130 treatment, MHD patients’ PSQI scores decreased from 10.7 ± 2.7 to 3.7 ± 2.0, and their insomnia remission rate and quality of life scores were significantly higher than those of the HD group.^43^^,^^44^ A quasi-experimental study of 40 MHD patients showed that after 8 weeks of HD+HA130 treatment (either weekly or biweekly), the total PSQI score significantly improved compared with the baseline score (mean total score reductions of –2.79 [*P* = 0.016] and –2.33 [*P* = 0.044], respectively).^45^

#### Resistant hypertension

**Recommendation 13:** We recommend a systematic assessment of blood pressure in MHD patients. After excluding secondary causes such as renal artery stenosis, primary aldosteronism, and pheochromocytoma, patients with inadequate blood pressure control should undergo optimized volume management and pharmacologic therapy, with further evaluation and management of resistant hypertension as needed (evidence quality: B; recommendation strength: strong; consensus level: 100%).

**Recommendation 14:** For MHD patients who are adequately dialyzed, have achieved their dry weight, and are taking three or more different classes of antihypertensive medications at the maximum or maximally tolerated dose, yet continue to have a predialysis systolic blood pressure > 160 mm Hg (1 mm Hg = 0.133 kPa), we recommend HA treatment once weekly or once every two weeks. Considering the potential adsorption of antihypertensive drugs by HA, we suggest adjusting the medication regimen based on blood pressure levels after the HA session (evidence quality: B; recommendation strength: strong; consensus level: 100%).

Resistant hypertension is a core factor leading to the sharp increase in cardiovascular events and mortality risk in MHD patients. Its pathophysiology is not only related to volume overload but is also critically driven by renin–angiotensin–aldosterone system overactivation.^46^ Before considering HA treatment, a systematic assessment must be conducted to exclude secondary causes, ensure that the patient has reached the ideal dry weight, and ensure that the patient has received a rational and sufficient multidrug antihypertensive regimen, thereby accurately diagnosing and managing resistant hypertension.

HD combined with once-weekly or biweekly HA130 treatment can significantly reduce SBP and DBP as well as renin, angiotensin II, and aldosterone levels in MHD patients with resistant hypertension; it can also significantly decrease the dosage of antihypertensive drugs needed.^47^^,^^48^ HD combined with alternating biweekly HDF and biweekly HA can improve blood pressure variability in MHD patients and reduce the risk of cardiovascular events.^49^

#### Restless Legs Syndrome

**Recommendation 15:** We recommend screening and conducting a systematic assessment for RLS in MHD patients, utilizing standardized scale tools to identify the condition and quantify symptom severity (evidence quality: C; recommendation strength: strong; consensus level: 100%).

**Recommendation 16:** For MHD patients with restless legs syndrome (RLS), particularly those with an International Restless Legs Syndrome Rating Scale (IRLS) score ≥ 11, we recommend once-weekly HA treatment (evidence quality: C; recommendation strength: strong; consensus level: 100%).

RLS, a common neurological complication in MHD patients, is characterized by discomfort that severely interferes with rest and sleep. It is not only a major cause of significantly reduced quality of life but also independently associated with increased cardiovascular risk and mortality. Its pathophysiology is thought to be related to dysfunction of the dopaminergic system and the accumulation of middle-molecular-weight uremic toxins.^50^ Routine screening using standardized scales such as the IRLS helps in the early detection and assessment of symptoms, providing a basis for timely intervention.

After 3 months of once-weekly HD+HA130 treatment, patients’ IRLS scores decreased from 13.5 ± 6.2 to 3.6 ± 1.4.^43^ Furthermore, this study identified that elevated serum iPTH and β_2_-MG levels are independent risk factors for the development of RLS in elderly MHD patients, suggesting that the clearance of these specific middle-to-large molecular toxins by HAHD directly mitigates RLS pathophysiology.^43^ The results from an RCT of 90 MHD patients showed that after once-weekly HAHD treatment, the IRLS score decreased from 25.13 ± 6.24 to 7.56 ± 1.21, with a treatment efficacy rate of 97.78%.^51^

#### Uremic Peripheral Neuropathy

**Recommendation 17:** We recommend screening and systematic assessment for uremic peripheral neuropathy in MHD patients, including symptom inquiry, physical examination, and necessary neurophysiological tests (evidence quality: C; recommendation strength: strong; consensus level: 100%).

**Recommendation 18:** For MHD patients with uremic peripheral neuropathy, particularly those presenting with distal numbness, paresthesia or sensory dullness, and reduced or absent tendon reflexes, or with electrophysiological involvement of ≥2 peripheral nerves, we recommend once-weekly HA treatment (evidence quality: C; recommendation strength: strong; consensus level: 100%).

Uremic peripheral neuropathy, one of the most common neurological complications of kidney failure, is driven by the accumulation of uremic toxins. It is a polyneuropathy characterized by length-dependent axonal degeneration that presents as distal symmetrical sensory–motor impairment. It can lead to progressive weakness, sensory loss, and functional impairment, severely affecting quality of life. Regular clinical assessment and neurophysiological testing can aid in early diagnosis and monitoring of disease progression, providing an objective basis for treatment decisions.^52^

Once-weekly HD+HA130 treatment effectively improves peripheral neuropathy symptoms in MHD patients; the sensory conduction velocities of the median, anterior tibial, and common peroneal nerves are all significantly accelerated, with efficacy comparable to that of peritoneal dialysis and superior to that of HD alone.^53^ HFHD, HDF, or once-weekly HD+HA130 can significantly increase patients’ sensory nerve conduction velocity, thereby ameliorating uremic peripheral neuropathy.^53^ The efficacy rate of HD+HA130 in ameliorating peripheral neuropathy symptoms reached 90.91%.^54^

#### Accumulation of PBUTs

**Recommendation 19:** We recommend screening and systematic assessment of PBUTs levels, focusing on indoxyl sulfate (IS) and p-cresyl sulfate (PCS), in MHD patients to identify high toxin burdens and related complication risks (evidence quality: B; recommendation strength: strong; consensus level: 100%).

**Recommendation 20:** For MHD patients with PBUT accumulation, particularly those with IS > 44 mg/L or PCS > 19 mg/L, we recommend once-weekly HA treatment (evidence quality: C; recommendation strength: strong; consensus level: 100%).

PBUTs, such as IS, PCS, and carboxymethyl-lysine, and their accumulation, can play key pathophysiological roles in driving the high incidence of cardiovascular events and high mortality rate in MHD patients through mechanisms such as the induction of oxidative stress, endothelial dysfunction, and systemic inflammatory responses.^55^^,^^56^ Although routine clinical testing for PBUTs is not yet widespread, dynamic monitoring of their levels can help accurately assess the toxin load and complication risk of individual patient, thereby guiding the formulation and optimization of PBUT clearance strategies, including HA; objectively evaluating the efficacy of blood purification technologies; and promoting standardization and quality improvement in dialysis therapy.^57^, ^58^, ^59^ Therefore, accelerating the development of real-time PBUT detection technologies suitable for routine clinical use and including key toxins such as IS and PCS in routine monitoring of MHD patients are recommended, which would help provide solid metrics for achieving precise management and evaluating efficacy.

Compared with HDF, an RCT involving 30 MHD patients showed that a single session of HD combined with a novel functionalized resin (pHA130) cartridge was more effective at removing IS (46.9% vs 31.8%, *P* = 0.044) and PCS (44.6% vs 31.4%, *P* = 0.003).^60^ An RCT of 20 MHD patients showed that compared with HFHD alone, a 4-hour HFHD session combined with HA130 significantly increased the clearance rate of carboxymethyl-lysine (64.7% vs 39.3%) without increasing the risk of clotting or other adverse events.^61^ Another multicenter prospective cohort study of 78 MHD patients showed that both 2-hour and 4-hour HD+HA130 treatments could effectively reduce IS and PCS levels, and the rates of reduction in IS and PCS levels after 4 hours of HA treatment were significantly higher than those after the traditional 2-hour HA protocol (IS: 56.17% vs 40.14%, *P* < 0.001; PCS: 47.39% vs 35.91%, *P* < 0.001).^62^

### Intensive treatment regimen

**Recommendation 21:** For MHD patients with severe, refractory complications that are unresponsive to routine treatment, we recommend short-term, intensive HA therapy (evidence quality: C; recommendation strength: strong; consensus level: 100%).

Short-term intensive HA treatment strategies should be individualized by increasing the treatment frequency or selecting adsorbers with higher efficacy for target toxins to alleviate severe symptoms rapidly and significantly. One study of 15 CKD-aP patients who were refractory to conventional treatment adopted short-term high-frequency HD+HA130 (3×/week); after the first treatment, the modified Duo’s pruritus score decreased from 24.9 ± 6.9 to 9.5 ± 6.2, and after 6 treatments, the score further decreased to 2.9 ± 2.0, indicating rapid symptom relief.^63^ A study of 17 patients with dialysis-related amyloidosis showed that 3×/week HD+Lixelle S15 treatment for 1 year significantly reduced serum β_2_-MG levels and increased activities of daily living scores.^36^ Furthermore, on the basis of the patient’s primary clinical problem, a cartridge with higher adsorption efficiency for specific toxins can be selected. For example, for patients with primarily β_2_-MG accumulation, a Lixelle-type β_2_-MG adsorption column may be prioritized; for the accumulation of other toxins, relatively specific adsorption materials such as CA-type cytokine adsorption columns (for inflammatory mediators) or pHA130-type cartridges (for PBUTs) can be used. The inflammation mitigation hypothesis proposed by Ronco et al^21^ suggests that an initial high-frequency (3×/week) intensive HA attack phase can rapidly reduce the body’s toxin burden and inflammation level; the subsequent transition to a 1×/week maintenance phase can still maintain a low inflammatory state and achieve long-term benefits. Once the desired therapeutic effect is achieved, a timely transition to the conventional maintenance frequency is warranted to balance efficacy and treatment burden.

### Long-term complication risk management

**Recommendation 22:** After contraindications are excluded, routine HA treatment is recommended for MHD patients to reduce the risk of long-term complications (evidence quality: B; recommendation strength: strong; consensus level: 100%).

A prospective multicenter cohort study of 4,623 MHD patients showed that, compared with the HD alone group, the HAHD group had significantly lower all-cause mortality (9.6% vs 16.1%); moreover, a survival analysis indicated that a 4×/month HA frequency was associated with the highest patient survival rate.^10^ Another meta-analysis including 12 studies (7 RCTs and 5 cohort studies) involving 1,331 MHD patients confirmed that compared with HD alone, HAHD significantly increased 1-, 2-, and 5-year survival rates. The potential factors for this survival benefit include the following: complementary clearance of metabolites, effective prevention and treatment of long-term dialysis complications, significant reduction in the levels of PBUTs and middle- to large-molecular-weight toxins, improvement in the state of microinflammation and renal anemia, protection of cardiac function, reduction in cardiovascular events, protection of residual kidney function, and improvement in gut dysbiosis.^11^ A cost-effectiveness analysis jointly conducted by the National Health Commission's Health Development Research Center and the National Drug and Health Technology Assessment Center showed that compared with patients treated with conventional blood purification, patients treated with HA resin HAHD could have their survival period extended by 10.5 years; moreover, the ICER was below the willingness-to-pay threshold of 3 times the per capita GDP, indicating economic viability.^64^ Similarly, the results from the Shanghai Health Development Research Center showed that for kidney failure patients, compared with HD alone, the HAHD regimen extended lifespan by 2.87 years, and its ICER was also below the 3× per capita GDP threshold, indicating good cost-effectiveness.^65^ According to the World Health Organization evaluation criteria, if an intervention’s ICER is less than 3 times the national per capita gross domestic product, it is generally considered cost effective.^66^ Therefore, these findings suggest that providing HA treatment for MHD patients in China has economic advantages.

## Treatment Modalities and Precautions

### Treatment mode

**Recommendation 23:** HA can be combined with various blood purification modalities, including LFHD, HFHD, and HDF (evidence quality: B; recommendation strength: strong; consensus level: 100%).

Clinical studies have confirmed that HA can be safely and effectively combined with LFHD, HFHD, and HDF to meet the individualized treatment needs of different patients.^7^^,^^67^ Moreover, combined therapy provides better clearance of PBUTs and middle- to large-molecular-weight toxins.^61^^,^^68^

### Connection Method

**Recommendation 24:** In combined therapy, we recommend connecting the cartridge in series, positioned before the filter (evidence quality: C; recommendation strength: strong; consensus level: 100%).

Placing the cartridge before the filter is the most commonly used and most studied connection method in clinical practice. This configuration is technically safe and feasible and does not significantly interfere with the pressure monitoring or operation of conventional HD or HDF.^14^^,^^69^

### Treatment Parameters

**Recommendation 25:** We recommend that key parameters (duration and blood flow) for combined therapy be individualized on the basis of a comprehensive assessment of therapeutic efficacy, safety, operational convenience, and patient condition (evidence quality: C; recommendation strength: strong; consensus level: 100%).

Each HA treatment session lasts 2-4 hours, and a specific duration should follow the product manual of the cartridge brand. Currently, most dialysis centers in China perform HA for 2.0-2.5 hours.^7^^,^^70^ For patients with high toxin loads or poorly controlled complications, the HA duration can be extended to match the entire HD session to achieve better clinical effects. Multiple studies have shown that if the patient tolerates it and monitoring is adequate, extending HA130 treatment to the full 4-hour HD session (in series) can significantly increase the clearance of various middle- to large-molecular-weight toxins and PBUTs and improve dialysis adequacy without increasing the risk of clotting or albumin loss.^45^^,^^61^^,^^62^^,^^67^ Currently, most HD sessions last 4 hours. The 4-hour full-session series model is simple to use and optimizes the nursing workflow.

When HA combined with HD or HDF is used, the blood flow rate is usually controlled at 150-250 mL/min. However, recent studies have indicated that higher blood flow is not only safe and feasible but also potentially provides additional benefits. For example, in an international study from Chile, the blood flow for HD+HA130 reached 350-400 mL/min; a related international study from Spain showed that this combined therapy’s blood flow reached (442 ± 18) mL/min.^61^^,^^67^ Furthermore, a multicenter study in Beijing showed that the average blood flow for 4-hour HD+HA130 was 302.64 ± 30.22 mL/min.^62^ Compared with 250 mL/min, this flow rate did not increase the risk of adsorbent shedding, nor did it significantly affect the adsorption rates of PBUTs and middle- to large-molecular-weight molecules; however, the clearance of small-molecule uremic toxins was significantly improved, without an increased risk of clotting or other adverse events. Although differences in average body weight, vascular access conditions, and clinical practice habits exist between Eastern and Western populations, multiple studies from China and abroad have consistently confirmed that increasing blood flow to ≥250 mL/min in combined HA therapy is safe and effective. Therefore, when clinical decisions are being made, patients should be assessed individually; for those with excellent vascular access and stable hemodynamics, higher blood flow rate (eg, ≥250 mL/min) can be considered to maximize blood purification efficiency while ensuring safety.

### Precautions

**Recommendation 26:** We recommend close monitoring of vital signs (blood pressure, heart rate), the extracorporeal circuit (clotting, leaks), and relevant laboratory results (blood counts, coagulation) during HA. Given the potential of HA to adsorb medications, we recommend adjusting dosing schedules on the basis of drug properties and patient status (evidence quality: C; recommendation strength: strong; consensus level: 100%).

To ensure the safety and efficacy of HA treatment, systematic patient assessment and close monitoring throughout the process are essential. Comprehensive pretreatment assessment helps in formulating individualized treatment plans and anticipating potential risks, whereas dynamic monitoring during treatment is key to timely detection and management of complications, ensuring patient safety. Given the broad-spectrum adsorption properties of HA, the plasma concentrations of many commonly used clinical drugs (such as some antibiotics, antihypertensives, and cardiovascular drugs) may be affected.^71^, ^72^, ^73^ Clinicians should conduct individualized risk assessments and manage concomitant medications on the basis of the pharmacokinetic properties of the specific drugs (eg, protein binding rate, molecular weight, and lipophilicity) and the adsorption characteristics of the perfusion cartridge used. Strategies may include adjusting the administration time to after the HA session, appropriately increasing the dose after the HA session, or using therapeutic drug monitoring (TDM) when available to guide dose adjustments, thereby ensuring therapeutic efficacy.^63^^,^^72^

**Recommendation 27:** HA is not advised for MHD patients with any of the following conditions: platelet count <60 × 10^9^/L, leukocyte count <4 × 10^9^/L, predialysis hypotension (<90/60 mm Hg), active bleeding, hemodynamic instability, or unstable vital signs. For patients already receiving HA, the treatment may need to be discontinued based on clinical judgment (evidence quality: C; recommendation strength: strong; consensus level: 100%).

The safety of HA treatment should be based on the patient’s stable basic physiological condition. For patients with specific high-risk factors, initiating HA treatment may pose more risks than benefits. Therefore, clinicians should make individualized decisions on the basis of a comprehensive assessment of the patient’s condition, focusing on the expected benefits, to determine whether to initiate or continue HA treatment.^14^^,^^69^

## Adverse Reactions and Management

### Biocompatibility issues

During the first 0.5-1.0 hours after starting HA, patients may experience chills, fever, chest tightness, and dyspnea. Immediate management includes oxygen, IV dexamethasone, etc. Blood tests may show a transient decrease in white blood cell or platelet counts. If symptoms do not resolve after management, HA treatment should be promptly discontinued.

### Adsorbent particle embolism

During treatment, if the patient experiences chest tightness, progressive dyspnea, hypotension, etc., clinicians should determine whether adsorbent particle embolism is present. Once an embolism occurs, treatment must be stopped immediately. Management includes oxygen or hyperbaric oxygen therapy, along with active symptomatic treatment.

### Air embolism

Patients may present with cough, chest tightness, sudden dyspnea, severe cyanosis, hypotension, or even coma. This is mainly due to incomplete removal of gas from the extracorporeal circuit before treatment, air return to blood, or insecure connections/damage in the bloodlines during treatment, allowing gas to enter the body. Once air embolism is confirmed, HA treatment must be stopped immediately. The patient should be placed in the left lateral decubitus position with the head down and feet up. Management includes 100% oxygen inhalation, mask oxygen or endotracheal intubation, and other types of cardiopulmonary support. If the air volume is large, right atrial or right ventricular puncture and aspiration may be performed if conditions permit.

### Coagulation dysfunction

Adsorption therapy may be accompanied by the adsorption of coagulation factors or significant platelet aggregation and activation during HA, leading to coagulation. Insufficient heparin dosage, insufficient blood flow, or low ambient temperature can all lead to coagulation. Close observation and timely management are required during treatment.

### Hypotension

Hypotension may occur because of reduced blood volume in the initial phase of HA, excessive ultrafiltration, or allergic reactions during treatment. Active prevention, early detection, rapid management, and appropriate volume expansion are required. HA treatment may need to be terminated early in patients who experience hypotension.

## Prospects

HA is becoming increasingly important for the comprehensive management of MHD patients. Its clinical value in clearing uremic toxins that are difficult to remove by conventional dialysis, improving clinical complications, improving quality of life, and optimizing long-term prognosis is supported by a growing body of high-quality research evidence. This consensus, based on the 2021 edition, systematically integrates recent key research findings, provides a treatment philosophy centered on complication risk management, further clarifies the indications for HA treatment, and suggests more diversified treatment protocols and broader application scenarios, aiming to maximize clinical benefits for MHD patients.

Future development and research in this field should focus on the following 3 avenues.

### R&D and application of new materials and technologies

Relying on continuous advances in materials science, the development and systematic evaluation of novel adsorbent materials should be prioritized. These materials should cover different PBUTs and inflammatory mediators and possess higher adsorption selectivity and capacity. In clinical practice, more precise HA treatment mode selection strategies should be explored on the basis of the characteristics of various materials.

### Advancement of clinical research

There is an urgent need for more rigorously designed, large-scale, multicenter RCTs with long-term follow-up to further clarify the optimal timing, frequency, duration per session, and total course of HA treatment. In particular, individualized treatment research that is based on a comprehensive assessment of the biochemical indicators, clinical symptoms, and prognosis of patients should be advocated to explore optimal treatment plans for patients at different risk stratifications.

### Exploration of expanded applications

The application potential of HA in special clinical scenarios, such as in the treatment of crush injuries from earthquakes, severe burns, and in public health events such as infectious disease pandemics, should be actively explored and systematically studied to provide more evidence for the expanded application of new HA technologies.
